# Implant Rehabilitation in Grafted Alveolar Clefts: Clinical and Radiographic Outcomes

**DOI:** 10.3390/dj14050287

**Published:** 2026-05-11

**Authors:** Tal Capucha, Ahmad Hija, Amir Bilder, Chaim Ohayon, Andrei Krasovsky, Dror Aizenbud, Adi Rachmiel, Omri Emodi

**Affiliations:** 1 Department of Oral and Maxillofacial Surgery, Rambam Health Care Campus, Haifa 3109601, Israel; capuchatal@gmail.com (T.C.); amirbilder@gmail.com (A.B.); haim.ohayon@mail.huji.ac.il (C.O.); jim83carter@gmail.com (A.K.); omri.emodi@gmail.com (O.E.); 2 Ruth & Bruce Rappaport Faculty of Medicine, Technion–Israel Institute of Technology, Haifa 3200003, Israel; d_aizenbud@rambam.health.gov.il; 3 Department of Orthodontics and Craniofacial Anomalies, School of Graduate Dentistry, Rambam Health Care Campus, Haifa 3109601, Israel

**Keywords:** alveolar cleft, dental implants, alveolar bone grafting, maxillary advancement, distraction osteogenesis, cleft lip and palate, implant survival, Bergland grading

## Abstract

**Objective:** Implant rehabilitation in grafted alveolar clefts requires a complex staged reconstructive approach, and a clinically important yet underexplored question is which patients require additional pre-implant block regrafting after primary grafting has already been completed. This single-centre retrospective cohort study aimed to evaluate whether a history of maxillary advancement is associated with a reduced likelihood of requiring pre-implant block regrafting (defined here as Re-graft 1), and to describe medium-term implant survival outcomes in a cleft implant cohort. **Methods:** Forty-two patients with Veau class III or IV cleft palate who underwent implant rehabilitation in grafted alveolar cleft sites between 2011 and 2023 were included. A total of 80 dental implants were evaluated at the implant level; analyses of primary grafting outcomes and the need for Re-graft 1 were performed at the patient level. Mean age at implant placement was 21.07 years (range, 17–38 years); mean follow-up was 83 months. Patients were categorised by maxillary advancement history: distraction osteogenesis (*n* = 11), orthognathic Le Fort I advancement (*n* = 9), or no advancement (*n* = 22). Bergland grades were assigned independently by two attending surgeons from postoperative radiographs. Implant outcomes were classified using the ICOI/Misch four-level scale (success, satisfactory survival, compromised survival, failure). Group comparisons used chi-square and Fisher’s exact tests. **Results:** Patients with any maxillary advancement history were significantly less likely to require Re-graft 1: 65.0% of patients with advancement did not require Re-graft 1, compared with 27.3% in the no-advancement group (Fisher’s exact *p* = 0.029; OR = 4.95). Overall implant survival was 93.75%; 58.75% of implants were classified as complete success, and 30.00% as satisfactory survival. **Conclusions:** In this observational, hypothesis-generating cohort, maxillary advancement history was associated with a lower likelihood of requiring pre-implant block regrafting. Implant rehabilitation showed favorable medium-term survival. These findings are limited by the retrospective single-center design, modest sample size, and absence of multivariable adjustment, and require confirmation in larger prospective studies with standardized regrafting criteria.

## 1. Introduction

Cleft lip and palate represents one of the most common congenital craniofacial anomalies, affecting approximately 1 in 700 live births worldwide. Comprehensive management requires a multidisciplinary and staged reconstructive approach spanning infancy through young adulthood, typically encompassing primary lip and palate repair in infancy, secondary alveolar bone grafting during the mixed dentition phase, orthodontic treatment, surgical correction of associated maxillofacial skeletal discrepancies where indicated, and definitive prosthetic rehabilitation as the final stage of this long-term reconstructive sequence [1,2]. Dental implants are increasingly recognized as the preferred restorative option for missing teeth in the cleft area, particularly for replacement of the congenitally absent lateral incisor, providing a fixed, functional, and esthetically satisfactory outcome when appropriate biological conditions have been established [1,2,3]. However, implant placement in the cleft area remains technically and biologically demanding: ridge morphology, alveolar bone volume, and soft tissue architecture are frequently compromised by the congenital defect, previous surgical interventions, and the inherent limitations of autogenous bone grafting at this site [1,2].

Previous studies on implant rehabilitation in cleft patients have primarily addressed implant survival rates, esthetic outcomes, and prosthetic reconstruction following grafting, collectively reporting favorable medium-term implant survival rates of 85–95% and supporting implant rehabilitation as a predictable option in appropriately selected patients [1,2,3,4]. More recently, structured decision-making frameworks for lateral incisor replacement in cleft patients have further emphasized that implant placement must be considered within the broader reconstructive continuum rather than as an isolated restorative event [5].

Secondary alveolar bone grafting, typically performed in the mixed dentition phase prior to eruption of the permanent canine, restores continuity of the maxillary dental arch, provides periodontal support for adjacent teeth, and creates the osseous foundation necessary for subsequent implant rehabilitation [6,7]. The optimal timing of this procedure is well established, and graft outcomes are influenced by multiple factors, including patient age, cleft morphology, graft material selection, and the quality of postoperative orthodontic management. Clinical and radiographic outcomes after secondary alveolar bone grafting are variable across centers and patient populations, and several methods have been proposed and validated for their assessment. Among these, the Bergland radiographic classification—which grades the height of the alveolar bone bridge relative to adjacent tooth root length on postoperative radiographs—remains one of the most widely applied tools in the cleft literature, despite acknowledged limitations in inter-rater reliability and its inability to capture volumetric bone changes [8,9]. Three-dimensional cone beam computed tomography (CBCT)-based volumetric assessment methods have been proposed to address these limitations and provide more objective quantitative data [10].

A clinically important intermediate question that has received comparatively limited attention in implant-focused cleft cohorts is which patients require additional pre-implant block regrafting after primary grafting has already been performed. This secondary augmentation step—defined in the present study as Re-graft 1—involves secondary or tertiary block grafting performed after primary grafting but before implant placement, and is clinically significant because it substantially increases treatment burden, donor-site morbidity, surgical complexity, and overall rehabilitation duration for patients who have already undergone complex multistage reconstruction. Green and Padwa [11] previously examined whether the timing of secondary alveolar bone grafting influences the need for additional bone augmentation prior to implant placement, representing an important precedent for this type of inquiry. However, whether a history of maxillary surgical advancement influences the subsequent need for pre-implant block regrafting has not been characterised in an implant-focused cleft cohort.

Maxillary advancement is frequently required in cleft patients because maxillary hypoplasia—a common sequel of early palatal repair due to scar tissue formation—may produce significant sagittal and vertical skeletal deficiency. This can be corrected by conventional Le Fort I osteotomy or by distraction osteogenesis, depending on the magnitude of deficiency and the overall treatment plan [12]. Beyond their primary function of correcting the skeletal discrepancy, both procedures reposition the entire maxilla anteriorly, thereby potentially altering the three-dimensional spatial relationship between the residual alveolar cleft site, the adjacent dentition, and the planned implant placement envelope. We hypothesise that in patients who have undergone maxillary advancement, this skeletal repositioning may favourably modify alveolar spatial relationships, effectively reducing residual alveolar bone deficiency at the cleft site to a degree that lessens the need for supplementary pre-implant block augmentation. In patients without such advancement, persistent maxillary hypoplasia and associated alveolar deficiency may more frequently necessitate additional block grafting to create an adequate implant site. Staged strategies specifically combining alveolar bone grafting with distraction osteogenesis to generate implantable bone volume in severely deficient maxillae have been previously described [12], providing biologic plausibility for this proposed relationship. However, direct evidence supporting this association in an implant-focused cohort is currently lacking.

The Bergland radiographic grade after primary grafting was included as a secondary outcome in this study to characterise the variability of initial graft results within the cohort, consistent with established reporting practice in the cleft literature [8,9], and to provide descriptive context for the biological complexity of the patient group.

The aim of this retrospective cohort study was therefore to evaluate, as an associative analysis, whether a history of maxillary surgical advancement was associated with a reduced likelihood of requiring pre-implant block regrafting (Re-graft 1) in patients who subsequently underwent implant rehabilitation in grafted alveolar cleft sites.

## 2. Materials and Methods

### 2.1. Study Design

This was a single-center retrospective cohort study conducted at the Department of Oral and Maxillofacial Surgery, Rambam Health Care Campus, Haifa, Israel, covering patients treated between January 2011 and December 2023. The study was designed and reported in accordance with the STROBE (Strengthening the Reporting of Observational Studies in Epidemiology) guidelines for observational research.

### 2.2. Participants

Inclusion criteria: Patients were eligible if they: (1) had a confirmed diagnosis of alveolar cleft associated with Veau class III or IV cleft palate; (2) had undergone secondary alveolar bone grafting followed by dental implant placement in the grafted cleft site at the study institution; and (3) had sufficient clinical and radiographic records available to reconstruct the complete staged treatment sequence, defined as the availability of operative records for all grafting and implant procedures, postoperative radiographs obtained at least 6 months after primary grafting, and clinical follow-up documentation at a minimum of 12 months after implant placement.

Exclusion criteria: Patients were excluded if: (1) implant rehabilitation was performed at a site other than the grafted alveolar cleft; (2) complete operative or radiographic records were unavailable for reconstruction of the treatment pathway; or (3) follow-up duration was insufficient to allow outcome classification according to the ICOI/Misch criteria.

The cohort was assembled by identifying all patients with alveolar cleft conditions who had undergone dental implant rehabilitation at the institution during the study period. Patients were individually assessed for clinical suitability prior to implant treatment, based on adequate oral hygiene, demonstrated capacity for regular follow-up attendance, and sufficient dental and skeletal maturity to support implant placement and long-term monitoring. This pre-implant clinical selection process is standard institutional practice and reflects the eligibility requirements applied to all patients considered for implant rehabilitation in the cleft population. The study, therefore, represents a consecutive series of all clinically eligible cleft patients who received implants within the defined study period, resulting in a final analytic cohort of 42 patients and 80 dental implants. All dental implants used in this cohort were Alpha-Bio Tec implants (Alpha-Bio Tec Ltd., Petah Tikva, Israel).

No formal a priori sample size calculation was performed because this was a consecutive retrospective cohort study in which all clinically eligible patients treated within the defined study period were included. Accordingly, the statistical analyses are exploratory and hypothesis-generating in nature rather than confirmatory.

The cohort consisted of 23 males (54.8%) and 19 females (45.2%), with a mean age at implant placement of 21.07 years (range, 17–38 years) and a mean follow-up of 83 months after implant placement. Baseline patient characteristics are summarised in Table 1.

Data were obtained through retrospective review of the institutional medical records and radiographic archive.

### 2.3. Staged Reconstructive Pathway

**Figure 1:**
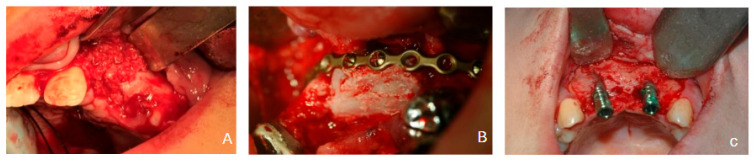
Representative clinical and radiographic sequence of the staged reconstructive pathway in alveolar cleft rehabilitation: (**A**) primary grafting, (**B**) Re-graft 1 when required prior to implant placement, indicated, and (**C**) implant placement with or without Re-graft 2.

The treatment pathway in this cohort was analysed as a staged reconstructive sequence comprising four principal phases: (1) primary alveolar bone grafting; (2) possible maxillary surgical advancement; (3) possible pre-implant block regrafting (Re-graft 1); and (4) dental implant placement with or without adjunct grafting at the time of implantation (Re-graft 2). Each stage is defined in the subsections below. A representative clinical and radiographic sequence of this staged pathway is illustrated in Figure 1.

#### 2.3.1. Primary Grafting

Initial alveolar cleft reconstruction was performed using autogenous bone graft harvested from either the iliac crest or the mandibular symphysis, according to the operative record and the judgment of the operating surgeon, which was guided by patient age, cleft morphology, required graft volume, and available donor bone. Radiographic outcome after primary grafting was assessed using the Bergland classification system [8], which grades interdental bone bridge height on postoperative radiographs relative to the adjacent tooth root length: grade 1 indicates normal or near-normal bone height (≥3/4 of root length); grade 2 indicates slight bone loss (<3/4 but ≥1/2 of root length); grade 3 indicates marked bone loss (<1/2 of root length); and grade 4 indicates absence of a visible bone bridge. Grading was performed using postoperative periapical and panoramic radiographs obtained at least 6 months after primary grafting, independently assigned by two attending oral and maxillofacial surgeons. In cases of disagreement, the final grade was determined by consensus discussion between both assessors. Formal inter-rater reliability statistics were not calculated given the small sample size; this represents a methodological limitation acknowledged in the Limitations section. The distribution of Bergland grades according to donor site is shown in Table 2.

#### 2.3.2. Maxillary Advancement

Patients were categorized into three groups according to their maxillary advancement history:

- Orthognathic surgery group: patients who underwent conventional Le Fort I osteotomy with anterior maxillary advancement;
- Distraction osteogenesis group: patients who underwent maxillary distraction osteogenesis;
- No advancement group: patients who did not undergo surgical maxillary advancement.

The indication for maxillary advancement was based on clinical and cephalometric assessment of skeletal discrepancy severity and was determined by the multidisciplinary cleft team independently of the present study.

#### 2.3.3. Re-Graft 1 (Pre-Implant Block Regrafting)

Re-graft 1 was defined as secondary or tertiary autogenous block grafting performed after the primary grafting stage and before dental implant placement, when clinical and radiographic evaluation at the pre-implant planning stage demonstrated insufficient alveolar bone volume to support implant placement. In this cohort, Re-graft 1 consisted of autogenous cortical block grafting harvested from either the iliac crest or the mandibular ramus. The decision to perform Re-graft 1 was made by the treating surgeon based on clinical judgment and radiographic assessment, rather than a prospectively defined volumetric threshold; this represents a limitation of the study design. The distribution of Re-graft 1 requirement according to maxillary advancement group is presented in Table 3.

#### 2.3.4. Re-Graft 2 (Adjunct Grafting at Time of Implant Placement)

Re-graft 2 was defined as any supplementary grafting material applied at the time of implant placement to augment the local implant site, using allograft material or additional autogenous bone, as documented in the operative records. The number of patients and implants that received Re-graft 2 is summarised in Table 1 and Table 4.

### 2.4. Outcome Measures

The primary analytic outcome was the requirement for Re-graft 1 before implant placement, evaluated at the patient level according to maxillary advancement history group.

The secondary radiographic outcome was the Bergland grade after primary grafting, used as a descriptive variable characterising the initial graft result across the cohort.

The secondary implant-related outcome was implant status at final follow-up, classified using the ICOI/Misch four-level quality scale as: success (no pain on function, no mobility, no suppuration, peri-implant bone loss ≤ 0.2 mm/year, and no gingival index > 1); satisfactory survival (minor complications present but implant functional and retained); compromised survival (significant complications present but implant retained); or failure (implant removed or non-functional), consistent with previously published implant outcome reporting in grafted cleft sites [1,3,4]. Implant outcomes are summarised in Table 4.

### 2.5. Follow-Up

Clinical and radiographic records were reviewed at the most recent available follow-up visit to document grafting history, implant clinical status, and survival. Mean follow-up after implant placement was 83 months.

### 2.6. Statistical Analysis

Descriptive statistics were used to summarise cohort characteristics. Categorical variables are presented as frequencies and proportions (*n*, %), and continuous variables as mean (range).

The association between maxillary advancement history and the need for Re-graft 1 was examined using a three-group chi-square contingency table analysis (distraction osteogenesis, orthognathic surgery, no advancement). Given the small subgroup counts in this analysis (expected cell counts < 5 in several cells), results were interpreted with caution and corroborated by Fisher’s exact testing. To address the primary clinical question more directly, a pre-specified grouped binary comparison was also performed: patients with any maxillary advancement (distraction osteogenesis or orthognathic surgery combined) versus patients with no maxillary advancement. This grouped comparison was analysed using Fisher’s exact test, and an odds ratio (OR) with 95% confidence interval (CI) was calculated. Statistical significance was defined as a two-sided *p*-value < 0.05. No multivariable adjustment was performed given the sample size; all analyses are therefore exploratory. Statistical analyses were performed using IBM SPSS Statistics, Version 31.0.1.0 (IBM Corp., Armonk, NY, USA).

## 3. Results

### 3.1. Cohort Characteristics

A total of 42 patients with grafted alveolar clefts underwent implant rehabilitation at the study institution between 2011 and 2023, resulting in placement of 80 dental implants. The cohort comprised 23 males (54.8%) and 19 females (45.2%), with a mean age at implant placement of 21.07 years (range, 17–38 years) and a mean follow-up of 83 months. Re-graft 2 at the time of implant placement was performed in 38 of 42 patients (90.5%) at the patient level and in 74 of 80 implants (92.5%) at the implant level. Complete baseline patient characteristics and Re-graft 2 use are presented in Table 1.

### 3.2. Radiographic Outcome After Primary Grafting

**Figure 2:**
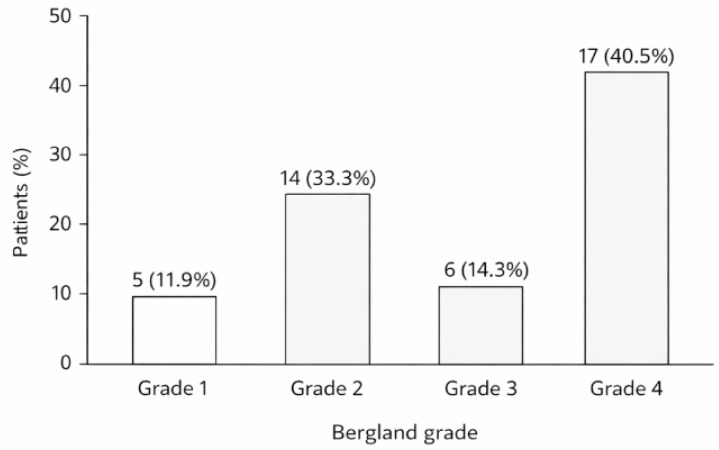
Distribution of Bergland grades after primary grafting.

Bergland grading after primary grafting revealed the following distribution across 42 patients: grade 1 (normal bone height) in 5 patients (11.9%), grade 2 (slight bone loss) in 14 patients (33.3%), grade 3 (marked bone loss) in 6 patients (14.3%), and grade 4 (no bone bridge) in 17 patients (40.5%). Primary grafting was performed using the iliac crest in the majority of patients and the mandibular symphysis in a smaller subset; the distribution of Bergland grades according to donor site is presented in Table 2. The high proportion of grade 4 outcomes (40.5%) reflects the severity of the cleft conditions in this cohort and is consistent with published reports of variable radiographic outcomes after secondary alveolar bone grafting in patients with Veau class III or IV cleft palate. The overall distribution of Bergland grades is illustrated in Figure 2.

### 3.3. Maxillary Advancement and the Need for Re-Graft 1

Among the 42 patients, 11 (26.2%) underwent distraction osteogenesis, 9 (21.4%) underwent Le Fort I orthognathic advancement, and 22 (52.4%) had no surgical maxillary advancement. The full distribution of the Re-graft 1 requirement by maxillary advancement group is presented in Table 3.

Within the distraction osteogenesis group, 8 of 11 patients (72.7%) did not require Re-graft 1, while 3 (27.3%) did. Within the orthognathic surgery group, 5 of 9 patients (55.6%) did not require Re-graft 1, while 4 (44.4%) did. Within the no-advancement group, only 6 of 22 patients (27.3%) did not require Re-graft 1, while 16 (72.7%) did.

**Figure 3:**
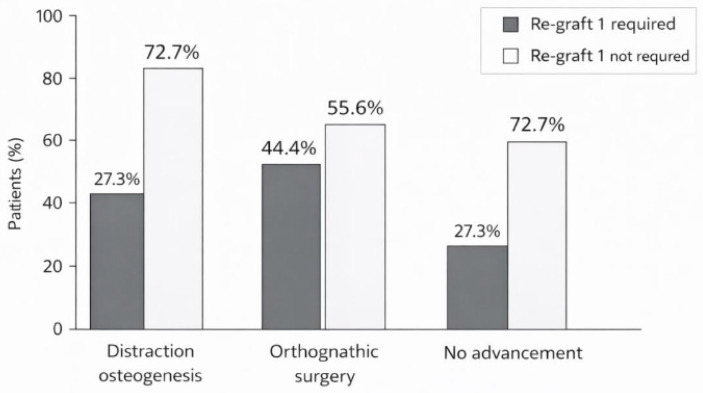
Requirement for Re-graft 1 according to maxillary advancement history (*p* = 0.029, Fisher’s exact test).

**Figure 4:**
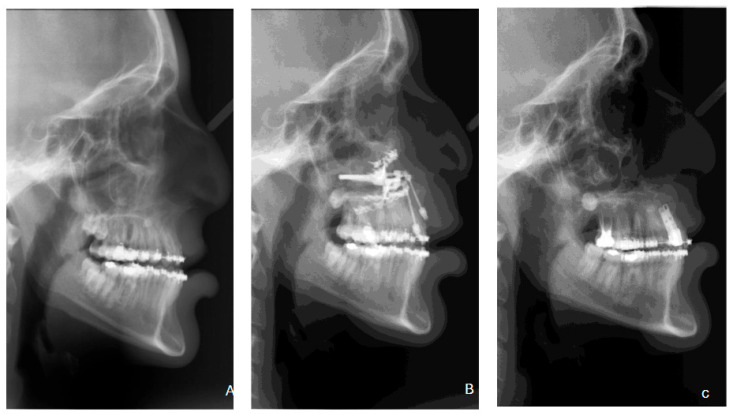
Representative maxillary distraction case showing cephalometric progression: (**A**) before advancement, (**B**) after advancement, and (**C**) after implant rehabilitation.

A statistically significant association between maxillary advancement history and Re-graft 1 requirement was identified across the three groups (chi-square, *p* = 0.037). In the primary grouped binary comparison, 13 of 20 patients with any maxillary advancement (65.0%) did not require Re-graft 1, compared with 6 of 22 patients (27.3%) in the no-advancement group. This association was statistically significant (Fisher’s exact *p* = 0.029; OR = 4.95, 95% CI: 1.33–18.41). These findings indicate that, within this cohort, a history of maxillary advancement was associated with a substantially reduced likelihood of requiring additional block regrafting before implant placement. The distribution of Re-graft 1 requirement by advancement group is illustrated in Figure 3, and a representative distraction osteogenesis case showing cephalometric and clinical progression is shown in Figure 4.

### 3.4. Implant Outcomes

At the implant level (*n* = 80), outcomes at final follow-up were classified as follows: success in 47 implants (58.75%), satisfactory survival in 24 (30.00%), compromised survival in 4 (5.00%), and failure in 5 (6.25%), yielding an overall implant survival rate of 93.75% (Table 4).

At the patient level, Re-graft 2 was performed in 38 of 42 patients (90.5%), and at the implant level it was recorded in 74 of 80 implants (92.5%). By outcome category, Re-graft 2 was present in all 47 successful implants (100%), all 24 implants with satisfactory survival (100%), 3 of 4 implants with compromised survival (75%), and none of the 5 failed implants (0%). The absence of Re-graft 2 in all failed implants is noted descriptively but should not be interpreted causally, given the small number of failures and the overall high rate of Re-graft 2 use across the cohort.

## 4. Discussion

The principal finding of this retrospective cohort study was that patients with a history of maxillary surgical advancement—whether achieved by distraction osteogenesis or conventional Le Fort I osteotomy—were significantly less likely to require pre-implant block regrafting (Re-graft 1) than patients without such a history. In the primary grouped binary comparison, the odds of not requiring Re-graft 1 were approximately five times greater in the advancement group than in the no-advancement group (OR = 4.95, Fisher’s exact *p* = 0.029). This finding is clinically meaningful because the need for additional block grafting before implant placement substantially increases the treatment burden, donor-site morbidity, and overall rehabilitation timeline for patients who have already undergone complex multistage cleft reconstruction.

Previous studies on implant rehabilitation in cleft patients have largely focused on implant survival, esthetic outcomes, and prosthetic reconstruction after grafting, with limited attention to the intermediate reconstructive decisions required to reach implant placement [1,2,3,4,5]. The most directly relevant precedent is provided by Green and Padwa [11], who examined whether the timing of secondary alveolar bone grafting influences the need for additional bone augmentation prior to implant placement at cleft sites. The present study extends this line of inquiry by examining maxillary advancement history as a distinct clinical factor within the same staged pathway. To our knowledge, the specific association between maxillary surgical advancement and the subsequent need for pre-implant block regrafting has not been previously characterised in an implant-focused cleft cohort, and this represents the primary novel contribution of the present work.

A plausible—though inherently speculative—mechanistic explanation for the observed association is that maxillary advancement favourably modifies local alveolar spatial relationships, reducing the residual bone deficiency at the cleft site to a degree that diminishes the need for supplementary pre-implant block augmentation. Both Le Fort I osteotomy and distraction osteogenesis reposition the entire maxilla anteriorly, potentially altering the three-dimensional relationship between the residual cleft defect, the adjacent dentition, and the spatial envelope required for implant placement. In patients with persistent maxillary hypoplasia and no skeletal correction, the alveolar relationships at the cleft site may remain less favourable, contributing to a higher frequency of Re-graft 1 requirement. Staged strategies combining alveolar reconstruction with distraction osteogenesis to generate implantable bone volume in severely deficient sites have been described [12], providing biological plausibility for this proposed relationship. However, this interpretation is emphasised as associative and inferential rather than causally supported by the current data, as the study was not designed to measure bone volumetric changes before and after maxillary advancement.

It is also important to acknowledge that the observed association may partly reflect selection effects rather than a true biologic mechanism. Patients selected for maxillary advancement may differ from those without advancement in clinically relevant ways not fully captured in the available dataset—including baseline skeletal severity, primary graft quality, cleft morphology, or the underlying trajectory of alveolar bone development—and these unmeasured differences may independently influence the subsequent Re-graft 1 requirement. Without multivariable adjustment or prospective standardisation of the Re-graft 1 indication, maxillary advancement history should therefore be regarded as a potentially useful clinical marker of reduced Re-graft 1 requirement rather than as a definitive causal determinant.

Bergland grading after primary grafting was included as a secondary descriptive outcome to characterise the variability of initial graft results within the cohort. The high proportion of grade 4 outcomes (40.5%) underscores the biological complexity of this patient group and the frequency with which primary grafting in severe cleft palate fails to produce a complete interdental bone bridge. Although one might expect the Bergland grade to predict the subsequent need for Re-graft 1—insofar as a poorer radiographic graft result at the primary stage may be associated with greater residual bone deficiency at the pre-implant assessment—the Bergland grade was not incorporated into the primary comparative analysis. This is because: (i) the Bergland grade was assessed at a time point preceding any maxillary advancement and does not reflect the bone volume available at the time of pre-implant planning; and (ii) the sample size was insufficient to support multivariable analysis including both Bergland grade and advancement history as covariates. Previous studies and systematic reviews have shown that alveolar bone grafting outcomes are influenced by timing, donor site, graft composition, and radiographic assessment method, and that direct comparison across studies remains challenging due to methodological heterogeneity [6,7,8,9,10,13]. Future studies should formally evaluate whether the Bergland grade independently predicts Re-graft 1 requirement, accounting for maxillary advancement history and other potential confounders.

The implant-related findings of the present study were consistent with previously published cleft implant cohorts and with pooled estimates from systematic reviews, which report implant survival rates in the range of 85–95% in grafted cleft sites [1,2,3,4]. An overall survival rate of 93.75% was observed in the present cohort. However, it is important to note that only 58.75% of implants were classified as full success under the ICOI/Misch criteria, while 30.00% were classified as satisfactory survival—a category that implies the presence of minor complications without implant loss. This distribution warrants a balanced interpretation: while the implant retention rate is favourable, a substantial proportion of implants in this complex population exhibit some degree of biological or functional compromise that requires ongoing clinical monitoring. This finding is consistent with the known challenges of implant rehabilitation in heavily reconstructed cleft sites and should not be overlooked in patient counselling regarding long-term maintenance expectations.

The broader reconstructive context further supports the plausibility of the staged pathway observed in this cohort. The literature describes a wide variety of grafting materials and techniques for alveolar cleft reconstruction, including autogenous corticocancellous and cortical block grafts, allograft materials, and biological adjuncts, with ongoing uncertainty regarding optimal approaches due to methodological heterogeneity across studies [14,15]. Block iliac grafting in older cleft patients has been associated with favourable radiologic and histologic healing [16], and CBCT-based studies suggest that donor site and graft composition may influence the quality of the reconstructed alveolar bone bridge [17]. More recently, cortical block grafting has specifically been described as a preparatory strategy for implant placement at cleft sites [18], and early complication profiles may vary according to graft design and composition [19,20,21,22]. Within this context, the need for Re-graft 1 in a subset of this cohort is not unexpected; the clinically relevant observation is that its frequency appeared substantially lower among patients who had previously undergone maxillary surgical advancement.

### 4.1. Clinical Significance

The findings of this study carry several potential implications for clinical practice in the multidisciplinary management of cleft patients undergoing implant rehabilitation. First, patients with a history of maxillary surgical advancement may require less extensive pre-implant surgical preparation than those without, potentially reducing the number of staged interventions, shortening the overall rehabilitation timeline, and decreasing the donor-site morbidity associated with additional block grafting procedures. Second, maxillary advancement history should be considered as one of several relevant factors in the pre-implant clinical assessment, alongside radiographic bone volume evaluation, soft tissue quality, periodontal status of adjacent teeth, and orthodontic staging. Third, the proportion of implants classified as satisfactory survival rather than full success underscores the importance of long-term implant monitoring and structured maintenance protocols in this patient population. Fourth, the high rate of Re-graft 2 use (90.5% of patients) confirms that adjunct grafting at the time of implant placement is frequently required even in sites deemed adequate for implant placement after the primary or secondary grafting stages, and this should be prospectively planned in treatment protocols for cleft implant rehabilitation.

### 4.2. Limitations

This study has several important limitations. First, the retrospective design limits the ability to control for treatment selection bias; patients selected for maxillary advancement may differ from non-advanced patients in clinically important ways not fully captured in the available dataset. Second, the sample size was modest (*n* = 42 patients; 80 implants), particularly after subdivision into three advancement subgroups, limiting statistical power for subgroup analyses and precluding multivariable adjustment. Third, no 95% confidence intervals were reported in the original analysis; these should be calculated and interpreted with caution given the sample size [note to authors: please insert CI for the OR]. Fourth, Bergland grading was performed independently by two attending surgeons without formal calculation of inter-rater reliability statistics (e.g., Cohen’s kappa coefficient), and the disagreement resolution method (consensus discussion) was not prospectively standardised. Fifth, the indication for Re-graft 1 was based on individual surgeon clinical judgment rather than a prospectively defined volumetric or radiographic threshold, introducing variability in decision-making that may have influenced group differences. Sixth, no multivariable analysis was performed to adjust for potential confounders such as cleft type and severity, Bergland grade after primary grafting, orthodontic status, patient age at grafting, or graft donor site. Seventh, the analysis of implant-level outcomes does not account for the non-independence of multiple implants within the same patient (clustering effect), which may affect the precision of implant-level estimates. These limitations indicate that the results should be interpreted as hypothesis-generating, and that the reported association should not be interpreted as evidence of causality.

## 5. Conclusions

In this single-centre retrospective cohort of patients with Veau class III or IV cleft palate, a history of maxillary surgical advancement—whether achieved by distraction osteogenesis or Le Fort I orthognathic surgery—was significantly associated with a lower likelihood of requiring pre-implant block regrafting (Re-graft 1). Implant rehabilitation demonstrated favourable medium-term survival (93.75%), supporting its role within the staged alveolar cleft reconstruction pathway, although the proportion of implants achieving full success (58.75%) emphasises the importance of long-term monitoring in this population. These findings are observational, non-causal, and hypothesis-generating in nature, and are subject to the limitations of a small retrospective single-centre design without multivariable adjustment. Nevertheless, maxillary advancement history may serve as a clinically relevant marker during pre-implant assessment and patient counselling in the cleft population. Future prospective multicentre studies with standardised pre-implant regrafting criteria based on validated volumetric imaging thresholds, formal multivariable regression analysis, and adequate sample sizes are needed to confirm this association and to contribute to evidence-based guidelines for implant rehabilitation planning in patients with alveolar cleft conditions.

## Figures and Tables

**Table 1:** Baseline patient characteristics and use of Re-graft 2 at implant placement (patient-level; *n* = 42).

Characteristic	Value
Patients, *n*	42
Implants, *n*	80
Sex, *n* (%)	
Male	23 (54.8)
Female	19 (45.2)
Age at implant placement, mean (range), years	21.07 (17–38)
Follow-up after implant placement, mean, months	83
Re-graft 2 at implant placement, *n* (%)	
Yes	38 (90.5)
No	4 (9.5)

**Table 2:** Distribution of Bergland grades after primary grafting according to donor site (patient-level; *n* = 42).

Bergland Grade	Total, *n* (%)	Iliac Crest, *n*	Symphysis, *n*
1	5 (11.9)	4	1
2	14 (33.3)	12	2
3	6 (14.3)	5	1
4	17 (40.5)	13	4
Total	42 (100)	34	8

**Table 3:** Requirement for Re-graft 1 according to maxillary advancement history (patient-level; *n* = 42).

Re-Graft 1	Distraction Osteogenesis (*n* = 11)	Orthognathic Surgery (*n* = 9)	No Advancement (*n* = 22)
Yes	3 (27.3)	4 (44.4)	16 (72.7)
No	8 (72.7)	5 (55.6)	6 (27.3)

Values are *n* (% within column). Three-group chi-square *p* = 0.037. Grouped binary comparison (any advancement vs. no advancement): Fisher’s exact *p* = 0.029; OR = 4.95 (95% CI: 1.33–18.41).

**Table 4:** Implant outcomes according to the ICOI/Misch implant success classification and use of Re-graft 2 at implant placement (implant-level; *n* = 80).

Outcome Category	Implants, *n* (%)	Re-Graft 2 at Placement, *n*/*N* (%)
Success	47 (58.75)	47/47 (100)
Satisfactory survival	24 (30.00)	24/24 (100)
Compromised survival	4 (5.00)	3/4 (75)
Failure	5 (6.25)	0/5 (0)
Overall	80 (100)	74/80 (92.5)
Implant survival (non-failure)	75/80 (93.75)	—

ICOI/Misch: International Congress of Oral Implantologists/Misch implant quality scale.

## Data Availability

The original contributions presented in this study are included in the article. Further inquiries can be directed to the corresponding authors.
